# Diabetes Influences the Fusion of AutophagosomesWith Lysosomes in SH‐SY5Y Cells and Induces Aβ Deposition and CognitiveDysfunction in STZinduced Diabetic Rats

**DOI:** 10.1002/alz70855_101510

**Published:** 2025-12-23

**Authors:** louyan Ma, Qiumin Qu

**Affiliations:** ^1^ Xi'an No.9 Hospital, Xi'an, ShaanXi, China; ^2^ The First Affiliated Hospital of Xi'an Jiaotong University, Xi'an, Shaanxi, China

## Abstract

**Background:**

Diabetes has been regarded as an independent risk factor for Alzheimer's disease(AD). This study investigates the effect of diabetes on autophagosome‐lysosome fusion using STZ‐induced diabetic rats and SH‐SY5Y cells, aiming to clarify the molecular mechanisms linking diabetes to Aβ deposition and AD pathogenesis.

**Method:**

In vivo, STZ‐induced diabetic rats were tested using the Open‐field and Morris water maze tests to assess cognitive function. Hippocampal ultrastructural changes were observed via electron microscopy, while Western blot and qRT‐PCR were used to measure Aβ, CTSD, CTSL, and Rab7 expression. In vitro, SH‐SY5Y cells were cultured under high glucose (HG) conditions with or without rapamycin (Rap) or 3MA. Autophagy was analyzed using electron microscopy and mRFP‐GFP‐LC3 adenovirus transfection. Lysosomal activity (ACP2) was measured via ELISA, and apoptosis was assessed using flow cytometry.

**Result:**

Diabetic rats showed impaired spatial memory in the Morris water maze test and increased hippocampal Aβ expression, with decreased CTSD and CTSL levels.

SH‐SY5Y cells in the HG group exhibited increased autophagosomes and enhanced autophagic flux but reduced lysosomal ACP2 activity.

Rab7, Cath L, and Cath D levels were lower in the HG group, impairing autophagosome‐lysosome fusion and Aβ clearance.

Rapamycin improved lysosomal function, reduced apoptosis, and restored Rab7, Cath L, and Cath D levels, while 3MA exacerbated these impairments.

**Conclusion:**

Diabetes disrupts autophagosome‐lysosome fusion, impairing Aβ clearance and lysosomal function, leading to increased apoptosis and cognitive dysfunction. These findings provide insights into the mechanisms of diabetes‐related AD and suggest potential therapeutic targets.

